# Outcome of CC7 transfer to median nerve and deep branch of ulnar nerve for patients with global brachial plexus avulsion

**DOI:** 10.3389/fmed.2025.1604280

**Published:** 2025-07-28

**Authors:** Yuzhou Liu, Hu Yu, Jingbo Liu, Jie Lao

**Affiliations:** ^1^Department of Hand Surgery, Huashan Hospital, Fudan University, Shanghai, China; ^2^NHC Key Laboratory of Hand Reconstruction, Fudan University, Shanghai, China; ^3^Shanghai Key Laboratory of Peripheral Nerve and Microsurgery, Shanghai, China; ^4^Institute of Hand Surgery, Shanghai, China; ^5^Institute of Hand Surgery, Fudan University, Shanghai, China; ^6^Department of Hand Surgery, Jing’an District Center Hospital, Fudan University, Shanghai, China

**Keywords:** CC7 nerve transfer, deep branch of ulnar nerve, median nerve, medial antebrachial cutaneous nerve, intrinsic muscle

## Abstract

**Introduction:**

As for global brachial plexus avulsion, the recoveries of intrinsic muscles are not satisfying after nerve transfers.

**Methods:**

A retrospective review of 31 patients treated with different CC7 transfers after GBPA was carried out. The modified group: CC7 transfer to median nerve and DBUN by medial antebrachial cutaneous nerve (MACN). The conventional group: CC7 transfer to median nerve. The mean follow-up period was 3 years.

**Results:**

After CC7 transfers, one patient got M3, one patient got M2 and two patients got M1 in abductor digiti minimi (ADM) in the modified group, while no patients had recovery in ADM in the conventional group. The recoveries of abductor pollicis brevis (APB) were similar between two groups. Motor unit potentials (MUP) of ADM and dorsal interosseous muscle (DIM) appeared in 4 and 2 patients respectively after surgery in the modified group. Nobody gained MUP of ADM or DIM in the conventional group. There were no statistical differences of MUP in APB, compound motor action potential (CMAP) in FDPI and FCR between two groups.

**Discussion:**

CC7 transfer to median nerve and DBUN by pedicled ulnar nerve and MACN could initially make intrinsic muscles regeneration in patients with GBPA, while not affect the recovery of median nerve.

## Introduction

**FIGURE 1:**
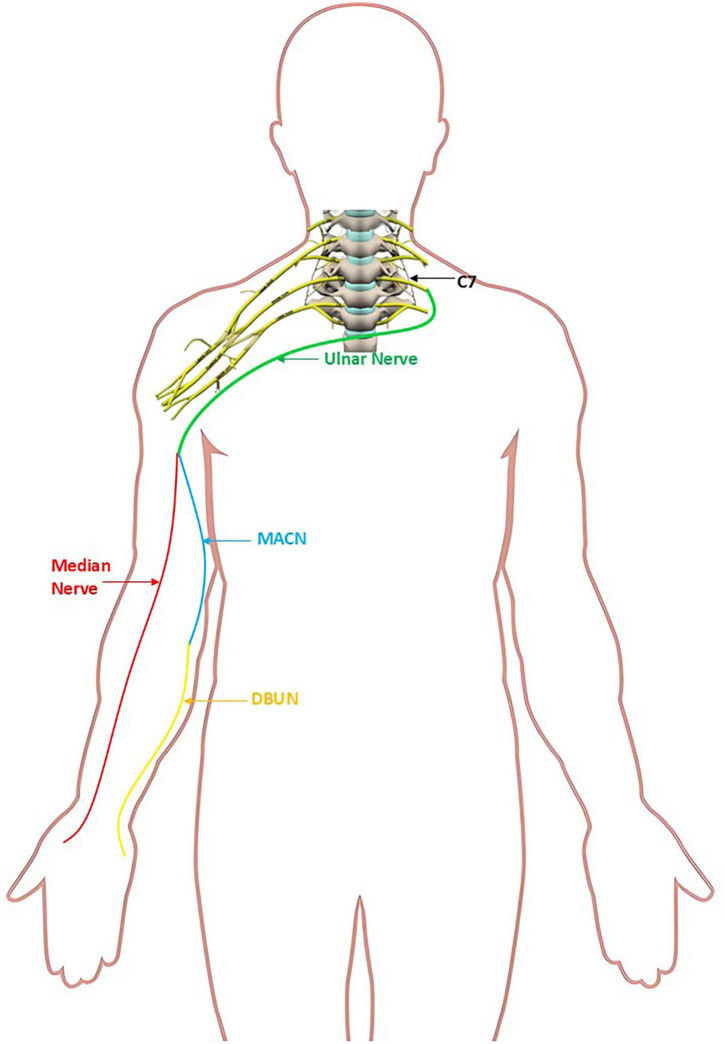
Schematic of contralateral cervical 7th (CC7) to ulnar nerve coaptation; ulnar nerve to medial antebrachial cutaneous nerve (MACN) and median nerve coaptation; MACN to deep branch of ulnar nerve (DBUN) coaptation.

As for global brachial plexus avulsion (GBPA), the recoveries of intrinsic muscles are not satisfying (1, 2) after various nerve transfers including phrenic nerve transfer (3), accessary nerve transfer (4), intercostal nerve transfer (5) and contralateral cervical 7th (CC7) nerve transfer (6), particularly because motor end plates irreversibly degenerate before the regenerated ulnar nerve axons are able to reinnervate the intrinsic muscles of the hand (7). Wang L et al. reported reinnervation of thenar muscle after repair of GBPA with CC7 root transfer in five cases (8). Yang X (9) reported fifty-three of 95 patients with GBPA exhibited motor unit potentials (MUP) recovery of abductor pollicis brevis (APB) after CC7 nerve transfer. CC7 could also be used to repair two or even three recipient nerves and achieved recoveries (10, 11). Due to the possibility of intrinsic muscle recovery, we modified the traditional CC7 transfer to both of the median nerve and deep branch of ulnar nerve (DBUN) (Figure 1) and conduct a clinical study to evaluate the effectiveness of this surgical approach for the regeneration of intrinsic muscles.

## Materials and methods

A clinical study was reviewed and approved by the institutional review board of Huashan hospital, Fudan university (2018416, date: 2018.10.30). The inclusion criteria included patients with GBPA, phrenic nerve, accessory nerve or intercostal nerve transfers completed, CC7 root was used to repair median nerve separately or both of the median nerve and DBUN. The diagnostic criteria of GBPA included (1) M0 occurred in all muscles which were dominated by C5-T1 nerve roots. (2) Preoperative and intraoperative electromyogram (EMG) detected that the upper limb muscles innervated by brachial plexus had no compound motor action potential (CMAP) or somatosensory evoked potential (SEP). (3) Intraoperative exploration showed C5-T1 nerve roots had avulsion. The exclusion criteria included diabetes, Volkmann contracture, fracture on the affected limb, and brain trauma.

According to inclusion and exclusion criterions, 36 patients were enrolled, including modified group (18 patients) and conventional group (18 patients).

All the patients were treated with two stage CC7 nerve transfers between 2018 and 2020. Phrenic nerve, accessory nerve and intercostal nerve transfers were used for the reconstruction of shoulder and elbow function. The sequence of the operations was phrenic and accessory nerve transfers simultaneously or accessory nerve transfer (phrenic nerve injury), the first stage of CC7 transfer (1 month later), intercostal nerve transfer (1 month later), the second stage of CC7 transfer (about 5 months later).

### Surgery

CC7 transfer was divided into two stages in both groups, with a 6-months interval between each stage.

#### Modified group

**FIGURE 2:**
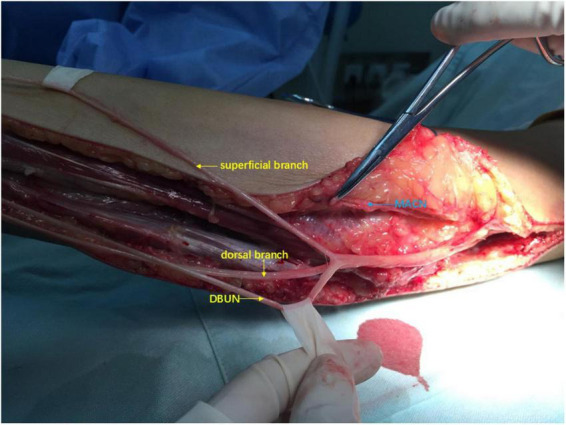
Intraoperative view: Deep branch of ulnar nerve (DBUN) was separated from superficial and dorsal branches of ulnar nerve from the wrist to forearm.

**FIGURE 3:**
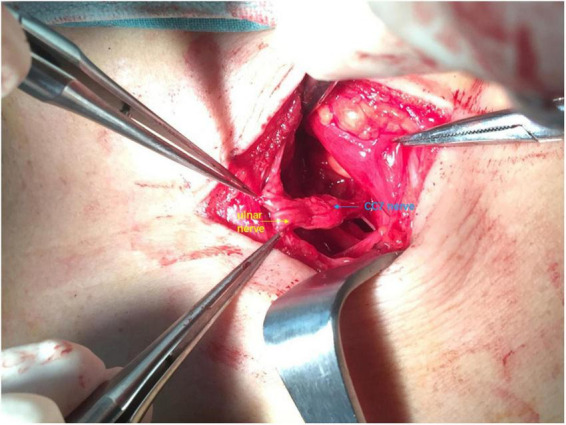
Intraoperative view: The distal end of ulnar nerve was sutured to the whole contralateral cervical 7th (CC7) root.

In the first stage, we used microscopic manipulation to separate DBUN from superficial and dorsal branches of ulnar nerve to minimize the disruption of blood supply in the superficial, deep and dorsal branches of ulnar nerve to the greatest extent (Figure 2). DBUN was cut off at its proximal end. The medial antebrachial cutaneous nerve (MACN) was exposed and transected. We sutured the distal end of MACN to the proximal end of DBUN under 2.5× magnification, using 8–0 microsuture. The vascularized ulnar nerve graft based on the superior ulnar collateral artery was harvested from the affected arm and delivered to the contralateral supraclavicular fossa through a subcutaneous tunnel. We sutured the distal ends of superficial and dorsal branches of ulnar nerve to the whole CC7 root under 2.5× magnification, using 8–0 microsuture (Figure 3). Every patient was immobilized by a head and arm brace to keep the head from turning to the affected side for 1 month.

**FIGURE 4:**
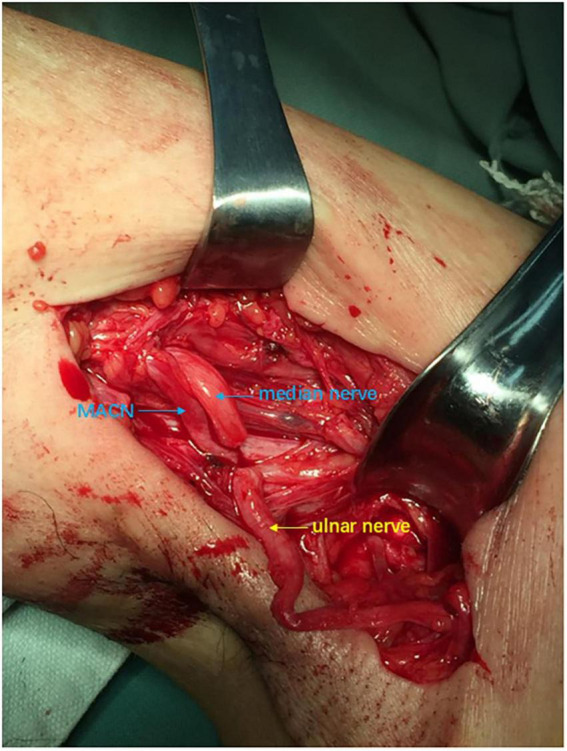
Intraoperative view: The ulnar nerve, medial antebrachial cutaneous nerve (MACN) and median nerve were exposed and cut off in the axillary position.

In the second stage, we exposed MACN, ulnar and median nerves near the retracing point of the ulnar nerve in the arm (Figure 4) and sutured the distal end of ulnar nerve to the proximal ends of MACN and median nerve under 2.5× magnification, using 8–0 microsuture. A sling was used to keep elbow flexion 90 degrees for 1 month.

#### Conventional group

In the first stage, the vascularized ulnar nerve graft based on the superior ulnar collateral artery was harvested from the affected arm and delivered to the contralateral supraclavicular fossa through a subcutaneous tunnel. We sutured the ulnar nerve to the whole CC7 nerve root under 2.5× magnification, using 8–0 microsuture. Each patient was immobilized by a head and arm brace to keep the head from turning to the affected side for 1 month.

In the second stage, we sutured the distal end of ulnar nerve to the proximal end of median nerve under 2.5× magnification, using 8–0 microsuture. A sling was used to keep elbow flexion 90 degrees for 1 month.

### Rehabilitation

We guided patients to start rehabilitation exercises and electrical stimulation therapy 1 month after second stage surgery. Perform 1000 functional exercises per day. Do wrist and finger flexion in the affected limb, while adducting the contralateral shoulder. Perform low frequency electrostimulation stimulation of each target muscle area to delay the fibrosis process twice a day, 20 min each time. Put the electrodes on the inner side of the affected upper arm near the axilla and thenar, hypothenar muscles. Vitamin B1, vitamin B6 and mecobalamin were suggested to take orally three times a day, 10 mg each time.

### Evaluation

The British Medical Research Council (MRC) grading system (12) and EMG examination were used to evaluate nerve regeneration. Return of muscle power of M3 or better was regarded as effective. EMG showed the condition of nerve regeneration, which included CMAP, MUP and no MUP.

### Statistical analysis

Comparisons between the modified and conventional groups were performed using Fisher’s exact test. The *p*-values were two-tailed and *p*-values < 0.05 were considered significant. All analyses were performed using Statistical Package for Social Sciences (SPSS) 19.0 software.

## Results

In the study, 3 and 2 patients lost in follow-up respectively in the modified and conventional groups. We followed up with 15 and 16 patients, respectively in the modified and conventional groups. The race of all patients was Han. In the modified group, 10 patients were males and one patient was female with a mean age of 29.0 years (range: 18–43 years) at the time of injury. The mean follow-up period was 3 years (range: 2.5–3.4 years). The delay in the first stage of CC7 transfer ranged from 2 months to 11 months. In the conventional group, 14 patients were males and 2 patients were female with a mean age of 35.2 years (range: 16–52 years) at the time of injury. The time from injury to surgery was from 2 weeks to 9 months, and the mean follow-up period was 3 years (range: 2.5–3.5 years). There was no significant difference between the two groups in terms of race, gender, age, time from injury to surgery or follow-up period.

In the modified group, motorcycle accidents accounted for GBPA in 11 patients. Two patients suffered falling from height. One patient had a machine traction injury. One patient suffered weight dropping on the shoulder. In the conventional group, motorcycle accidents accounted for GBPA in 10 patients. Other road accidents included a pedestrian accident. Three patients had machine traction injuries in the upper limbs. One patient suffered weight dropping on the shoulder and one suffered falling from height.

### MRC grading

All of the muscle strengths were M0 in the affected limb with GBPA preoperatively in the two groups. The tested muscles were the main targets of the recipient nerves by CC7 transfer. Median nerve: flexor carpi radialis (FCR), flexor digitorum profundus of index finger (FDPI) and APB. DBUN: abductor digiti minimi (ADM).

Table 1 showed that as for ADM, one patient got M3, one patient got M2 and two patients got M1 in the modified group, while no patients had recovery in the conventional group. APB recovered muscle strength of M3 in one patient, M2 in two patients, M1 in one patient and M0 in 11 patients in the modified group. One patient recovered strength of M3 and M2 in APB, three patients recovered strength of M1, whereas 11 patients had no recoveries in APB in the conventional group. The effect rate of FDPI was 46.7% in the modified group, while 43.8% in the conventional group. The effect rate of FCR was 53.3% in the modified group, while 50% in the conventional group. There were no significant differences in effect rates of FDPI and FCR between the two groups.

**TABLE 1:** Comparison of muscle strength in different muscles between two groups.

Muscle strength (MRC)	ADM		APB		FDPI		FCR	
	Modified group	Conventional group	Modified group	Conventional group	Modified group	Conventional group	Modified group	Conventional group
M4	0	0	0	0	3	2	3	3
M3	1	0	1	1	4	5	5	5
M2	1	0	2	1	1	0	2	0
M1	2	0	1	3	4	3	2	3
M0	11	16	11	11	3	6	3	5

### Electromyography

A MUP shown in EMG was regarded as a muscle regeneration signal and a CMAP was regarded as an effective recovery of motor function. No MUP were showed in the intrinsic muscles, FDPI or FCR in the affected limb in the two groups before surgeries.

After CC7 transfers, MUP of ADM and dorsal interosseous muscle (DIM) appeared in 4 and 2 patients respectively in the modified group. One of them recorded CMAP in ADM and DIM after stimulating DBUN at the wrist. Nobody gained MUP of ADM or DIM in the conventional group. 4 and 5 patients gained MUP in APB in the modified and conventional groups, respectively. Among them, one patient gained CMAP in APB in the modified and conventional groups, respectively. No statistical difference of MUP in APB existed between the two groups. As for FDPI, EMG showed CMAP appearing in 7 (46.7%) patients and MUP in 4 patients in the modified group. 7 (43.8%) patients had CMAP and 3 patients had MUP in the conventional group. In each group, 8 patients had CMAP and 3 patients had MUP in FCR. There were no statistical differences of CMAP in FDPI and FCR between the two groups (Table 2).

**TABLE 2:** Comparison of Motor unit potentials (MUP)/compound motor action potential (CMAP) in different muscles between two groups.

Group	APB		ADM		DIM		FDP of index finger		FCR	
	No MUP	MUP	No MUP	MUP	No MUP	MUP	No CAMP	CMAP	No CAMP	CMAP
Modified group	11	4	11	4	13	2	8	7	7	8
Conventional group	11	5	16	0	16	0	9	7	8	8

In the study, the main influencing factors of surgical efficacy included gender, age, time from injury to surgery and follow-up period. Multivariate regression analysis showed that there was no correlation between age, gender, time from injury to surgery or follow-up period and efficacy (MRC and EMG results).

### Complication

A total of 31 patients experienced paresthesia on the thumb, index, and middle pulp of the donor hand within 3 months after surgery and the sensory deficit completely recovered spontaneously in all patients when they followed up.

## Discussion

In the study, four patients experienced a recovery in muscle strength of ADM in the modified group and no patients had recovery of ADM in the conventional group. The MUP of ADM and DIM innervated by DBUN appeared in 4 and 2 patients respectively in the modified group. However, there is no MUP present in ADM or DIM in any patient in the conventional group, which were consistent with the MRC results. Because DUBN and superficial branches of the ulnar nerve, along with the dorsal branch were used as bridging nerves. This indicated that the modified CC7 transfer could regenerate the intrinsic muscles innervated by DBUN. The recoveries of APB muscle strength were similar between the two groups. Due to the small quantity, effective statistical comparison of ADM and APB could not be conducted. There were no significant differences in effect rates of FDPI and FCR muscle strength between the two groups. There were no statistical differences of MUP in APB and CMAP in FDPI and FCR between the two groups, which indicated compared with CC7 transfer to median nerve, CC7 transfer to both median and DBUN did not affect the recovery of median nerve.

We evaluated the effect of CC7 transfer for more than 6 months delay patients, including one 11 months delay patient in the modified group and one 9 months delay patient in the conventional group. Neither of the two patients had muscle strength or MUP appearance of intrinsic muscles, while M3 and CMAP existed in FCR and FDPI in both patients. Considering that due to the prolonged time from injury to surgery and intrinsic muscles were small, fibrosis of intrinsic muscles may result in their inabilities to recover.

The conventional CC7 transfer uses the whole ulnar nerve in the affected limb to connect the CC7 with target nerves, which makes it impossible to reserve the affected ulnar nerve and loses the possibility of recovery in the intrinsic muscles innervated by DBUN. We designed a modified CC7 nerve transfer: CC7–dorsal and superficial branches of the ulnar nerve–median nerve + MACN–DBUN, which reserved the DBUN and maximized the possibility of intrinsic muscles regeneration. DBUN could generally be non-invasively separated to about 8 cm above the wrist and the distal end of MACN in the forearm was generally located in the upper one-third of the forearm. Therefore, DBUN required further invasive micro-separation toward the proximal end for suturing to MACN. Hong GH (13) carried out an anatomy study. In 10 cadavers, the distal end of MACN and the proximal end of DBUN were close to each other. The ratios of MACN to DBUN in axon numbers were 0.61:1 on the left side and 0.65:1 on the right side. The ratios of ulnar nerve to the sum of median and MACN in axon numbers were 0.94:1 on the left side and 0.93:1 on the right side. It was feasible to suture MACN to DBUN, ulnar nerve to median nerve and MACN, based on the principle that the axon number in the donor nerve should equal to at least 30% of that in the recipient nerve (14).

Using the entire C7 for neurotization, the vascularized ulnar nerve graft, performing staged surgery, accurate microsurgical suture, postoperative regular electrical stimulation therapy and extensive functional exercises were our strategies for stimulating as many axons as possible to reach the target muscles. But even so, we still could not guarantee enough axons reach the motor end plates of the forearm muscles and intrinsic muscles through three neurorrhaphy sites. The study results showed that only a portion of patients achieved regeneration of forearm and intrinsic muscles and the recovery effects of intrinsic muscles were inferior to those of flexor muscles. But previously, the recovery of intrinsic muscles innervated by the ulnar nerve after total brachial plexus avulsion was zero, which was a fact. This modified surgery did produce therapeutic effects on the regeneration of intrinsic muscles in some patients.

Chen Xi (15) conducted an experimental study. She attempted to improve intrinsic function recovery by preserving DBUN and reanimating it with the anterior interosseous nerve after cC7 transfer for the GBPA rats. The results showed the modified CC7 transfer could potentially improve intrinsic function recovery without affecting median nerve recovery. But there were no clinical reports on this surgical method yet. Wang SF (16) proposed a procedure of direct CC7 nerve transfer to the lower trunk in the affected limb through the prespinal route without a graft. Among 20 patients, 11 patients were performed the humeral shorten osteotomy for reducing nerve suture tension. Only one patient recovered to M3 and one recovered to M2 muscle strength in ADM. Li YW (17) used hemi-CC7-to-lower trunk (LT) transfer through the modified prespinal route combined with clavicle osteotomy at 45° shoulder abduction. 63% patients achieved ≥ M3 hand grip recovery, but intrinsic muscle recovery was not reported. One patient experienced symptomatic phrenic nerve injury and the long-term donor-site complication rate was 2.6%. These CC7 nerve transfer to the lower trunk procedure were more complicated and riskier than our modified CC7 transfer.

This study had some limitations. The small sample size resulted in some statistical comparisons being unable to be conducted. This study belonged to a single-center clinical study. The reason for the bias in the single center study was not due to the source of patients, who came from all over the country, but rather because the surgery required skilled and meticulous surgical techniques, which our surgeons possessed. If surgeons in other hospitals were new to it, there might be insufficient efficacy due to lack of familiarity. In the study, we guided patients to do rehabilitation exercises, electrical stimulation and take oral neurotrophic drugs, but we could not accurately count and determine the compliance of patients with rehabilitation exercise and physical therapy after returning home, which might induce some potential bias. The mean follow-up period was 3 years in each group. Due to the growth rate of peripheral nerves being about 1 mm per day (18), the annual growth distance is around 36 cm generally. The length from the C7 anastomosis on the healthy side to the intrinsic muscle of the affected hand is approximately 70–100 cm (depending on the patient’s arm extension length). Therefore, 3 years was relatively short for the intrinsic muscle recovery, so this study was an early-stage clinical study. In the future study, we will conduct a 5 years-long-term follow-up and add fine motor skill assessments.

## Conclusion

This early-stage clinical study indicated CC7 nerve transfer to median nerve and deep branch of ulnar nerve by pedicled ulnar nerve and medial antebrachial cutaneous nerve could initially make the intrinsic muscles regeneration in patients with GBPA, while not affect the recovery of median nerve. Although early follow-up results showed this surgery did not make significant recovery of intrinsic muscles for most patients, it did produce therapeutic effects on the regeneration of intrinsic muscles in some patients, who were given the opportunities for recovering hand functions.

## Data Availability

The raw data supporting the conclusions of this article will be made available by the authors, without undue reservation.
